# Does Reverse Shoulder Arthroplasty Improve Function and Quality of Life in Patients With Rotator Cuff Tear Arthropathy?

**DOI:** 10.7759/cureus.42896

**Published:** 2023-08-03

**Authors:** Gökhan İlyas, Ercument Egeli

**Affiliations:** 1 Orthopedics and Traumatology, Uşak University, Uşak, TUR; 2 Orthopedics and Traumatology, Uşak Esme State Hospital, Uşak, TUR

**Keywords:** physical activity, quality of life, rotator cuff tear arthropathy, total shoulder replacement, shoulder prosthesis

## Abstract

Background

This study aims to determine the change in functional capacity and quality of life in patients who underwent reverse shoulder arthroplasty (RSA) due to rotator cuff tear arthropathy (RCTA).

Methodology

A total of 89 patients who underwent RSA due to RCTA between 2016 and 2022 were included in the study, as examined by the senior author. The Constant-Murley Score (CMS) was used for functional assessment, whereas the Short Form-36 (SF-36) was used for quality-of-life evaluation. The assessment scores before and after the surgery were compared to the reference values of CMS and SF-36 scores, as determined by reference studies, based on age groups. The change in functional capacity and quality of life with RSA was calculated. In addition, the impact of age and body mass index (BMI) on the results was examined.

Results

No differences were found in demographic data except for BMI (p = 0.026). CMS did not reach the reference values of a normal shoulder during the 12-month postoperative period. However, a significant increase of 156% in comparison to the preoperative values was detected (p < 0.001). In the postoperative period, there was a statistically significant improvement in SF-36 scores compared to preoperative scores, except for social functioning (p = 0.099) and vitality (p = 0.255) (for all other parameters, p < 0.001). In patients under the age of 65 years, all parameters except for physical functioning, physical role, and social functioning statistically reached the reference values. A decrease in CMS scores was noted with an increase in BMI, and the negative correlation further increased in the postoperative period (preoperative: r = -0.274, 12-month postoperative: r = -0.476).

Conclusions

RSA performed for RCTA leads to an improvement in functional outcomes and quality of life. Although there was a considerable improvement after surgery for CMS compared to preoperatively, it was observed that normal shoulder reference values ​​could not be reached. For SF-36, it was observed that it reached normal shoulder reference values, especially in patients over 65 years of age.

## Introduction

Rotator cuff tear arthropathy (RCTA) was defined by Neer et al. in 1983 as the collapse of the humeral head and glenohumeral joint disorganization resulting from massive rotator cuff tears [1]. Reverse shoulder arthroplasty (RSA), which was first described in 1970, is widely used in the treatment of not only RCTA but also in the management of multi-fragment fractures involving the anatomical neck of the humerus [2].

The Constant-Murley scale (CMS), which is commonly used for shoulder functional scoring, helps evaluate pain, strength, range of motion, and daily activities. It was initially described in 1987 and revised in 2008 [3]. In 2016, the CMS was translated into Turkish and validated by Çelik [4]. Subjective measurements can reach a maximum score of 35, while objective scores can reach a maximum score of 65, with higher scores indicating increased functional capacity (the maximum total score is 100). In this study, the Turkish translation of CMS was used for the functional assessment of patients.

Short Form-36 (SF-36) is commonly used in general health screening [5,6]. It was translated into Turkish and validated by Koçyiğit et al. in 1999 [7]. The SF-36 scale, consisting of 36 questions and eight subdomains, assesses physical components (physical functioning, physical role, bodily pain, general health) and mental components (mental health, emotional role, social functioning, vitality). A high score indicates a higher quality of life (the maximum score is 100). In this study, the Turkish translation of SF-36 was used for the assessment of quality of life.

It is well-known that CMS and SF-36 scales are frequently used in the evaluation of shoulder disorders [8-15]. However, in a study conducted by Castricini et al. [16], which examined patients with osteoarthritis, massive rotator cuff tear, and RCTA together, the postoperative health-related quality of life and functionality were examined after RSA. Apart from this study, which included 13 patients with RCTA, no similar study was found in the literature. Considering that 13 patients are insufficient to reach a definite conclusion, the main hypothesis of this study was to increase the power and the sample size with more patients. This was supported by the post hoc analysis made at the end of the study.

The primary goal of this study is to determine whether RSA performed for RCTA, which is becoming increasingly common, increases the functional status and quality of life of patients.

## Materials and methods

Between 2015 and 2023, a total of 173 patients who underwent RSA by the lead researcher were retrospectively screened after obtaining ethical approval (Uşak University Clinical Research Ethics Committee, date: 12/07/2023, decision number: 153-153-10). Informed consent was obtained from all patients.

**Figure 1:**
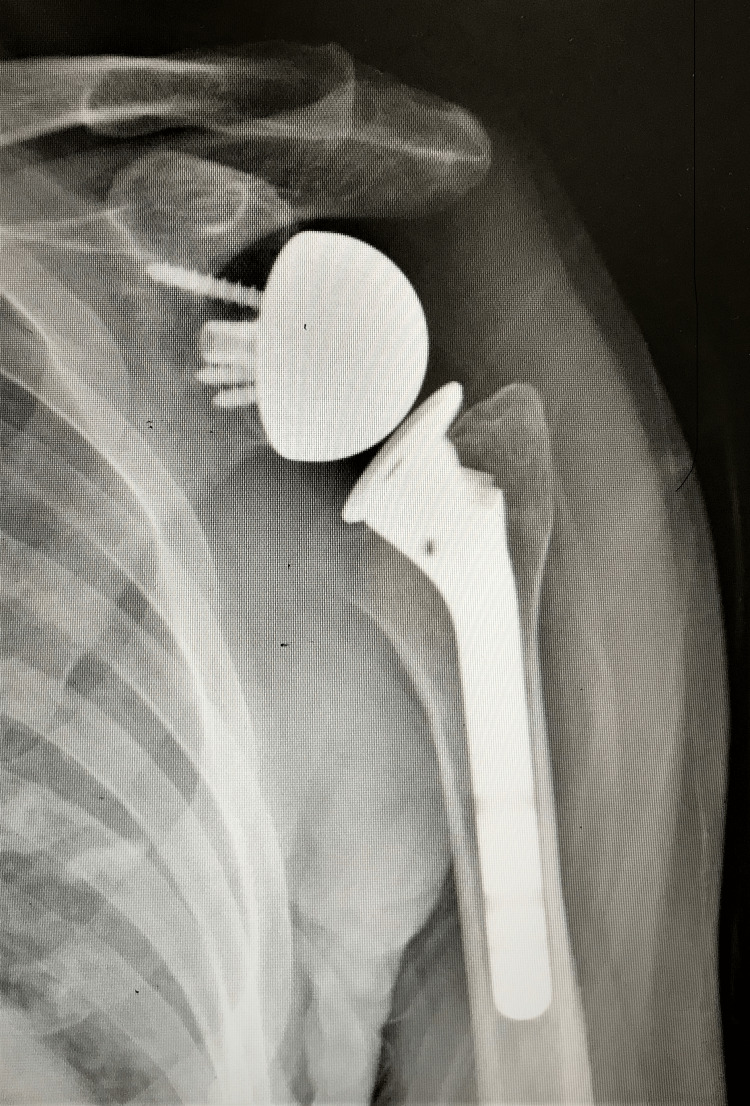
Postoperative 12th-month X-ray of a patient included in the study.

**Figure 2:**
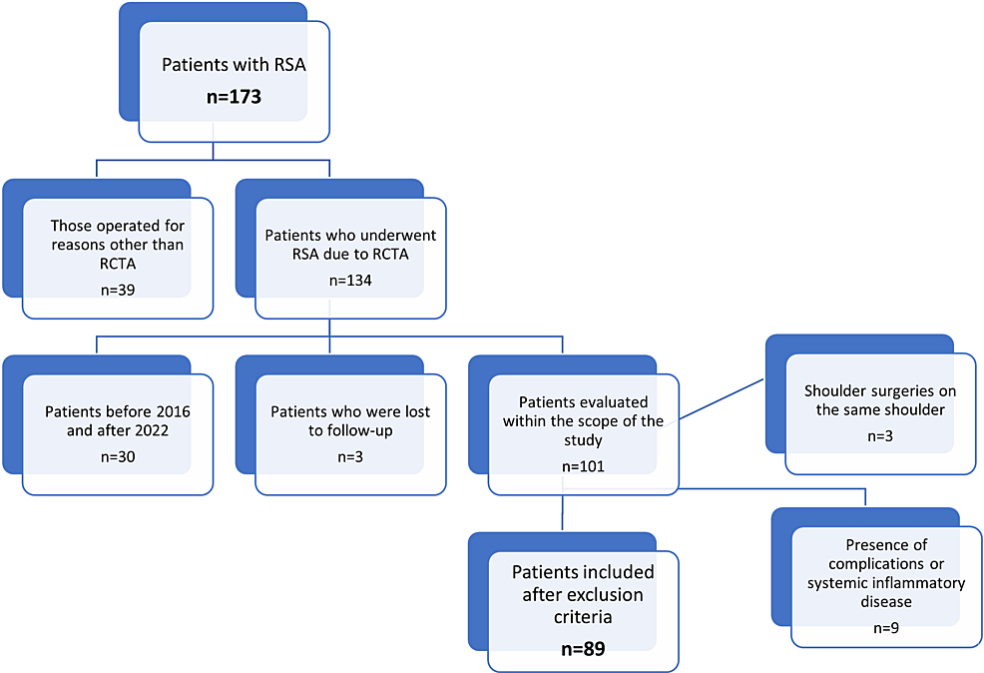
Flowchart of cases. RSA: reverse shoulder arthroplasty; RCTA: rotator cuff tear arthropathy

Patients who underwent RSA due to RCTA without a previous history of operation on the same shoulder by the lead author between 2016 and 2022 were included in the study (n = 89, 51%) (Figure 1). Age, gender, and dominant extremity use were not differentiated during inclusion, and demographic data were evaluated. In our clinic, the Hamada classification [17] was used in the evaluation of RCTA. Using the Hamada classification, patients between stages 1 and 5 were included in the study. There were eight patients in stage 1, 12 in stage 2, 21 in stage 3, 14 in stage 4a, 15 in stage 4b, and 19 in stage 5. Patients operated on before 2016 (n = 11) were excluded because they were operated on before the translation of the CMS into Turkish. Moreover, patients operated on after 2022 (n = 19) were excluded because the 12-month follow-up was not completed. Additional exclusion criteria included patients who were lost to follow-up (n = 3), those who underwent surgery for reasons other than RCTA (n = 39), those who experienced complications during or after the surgery (n = 3), those with a history of previous arthroscopic or open shoulder surgeries on the same shoulder (n = 3), and those suffering from systemic inflammatory diseases (n = 6) (Figure 2).

The CMS values obtained preoperatively, at the sixth-week evaluation, at the sixth-month evaluation, and at the 12th-month evaluation, as well as the SF-36 values obtained preoperatively and at the 12th-month evaluation, along with demographic data, were obtained from the archived patient files. By comparing the data obtained before and after the surgery, the changes in the functional capacities and quality of life of the patients were calculated after RSA operations performed due to RCTA. In addition, the data were further analyzed by grouping patients according to age to investigate whether age had an impact on the outcomes. In addition, reference normal values obtained from the literature were compared with the results of this study, and it was determined whether normal reference values could be reached [18,19]. Reference values for the CMS were determined in the original study as decathletes [18]. As four patients in this study were in their sixth decade, 53 in the seventh decade, and 32 in the eighth decade, CMS results were evaluated in two age groups (seventh decade, n1 = 53; eighth decade, n2 = 32) (because statistical calculations could not be performed for patients in the sixth decade, an additional group was not created. However, they were included in the overall calculations). In the study conducted by Demiral et al. [20], which indicated the reference values for SF-36 in the Turkish population, the population was evaluated into the following three groups: 18-44 years old, 45-64 years old, and 65 years and older. In this study, as there were no participants under the age of 45, the SF-36 results were evaluated in two age groups (45-64 years, n1 = 28; 65 years and older, n2 = 61). In addition, the effect of body mass index (BMI) on the scale values was examined.

Statistical analysis

The statistical analysis was performed using SPSS version 24 (IBM Corp., Armonk, NY, USA). After conducting the Shapiro-Wilk test for normality analysis, in cases where normal distribution was not observed, the Mann-Whitney U test and Spearman correlation analysis were used. In cases where normal distribution was observed, independent t-test and Pearson correlation analysis were used. A summary independent-sample t-test was used for comparing the study data with the literature data. A p-value less than 0.05 was considered statistically significant. The correlation coefficient (r) used in correlation analyses was interpreted as follows: values <0.3 indicated a very weak correlation, 0.3-0.5 indicated a weak correlation, 0.5-0.7 indicated a moderate correlation, and >0.7 indicated a strong correlation. Post-hoc analysis was performed using the G*power 3.1.9.7 program (Heinrich-Heine-Universität Düsseldorf, Germany).

## Results

The mean age of the 89 patients included in the study was 67.96 ± 4.91 years (range = 57-77 years). The mean age of female patients was 66.95 ± 5.23 years (range = 57-76 years) (n = 68), while the mean age of male patients was 68.26 ± 4.79 years (range = 58-77 years) (n = 21) (p = 0.286). Out of the 89 patients, 60 (67%) underwent surgery on their dominant extremity. When evaluated based on age, there was no statistically significant difference (p = 0.428). BMI was 28.44 ± 2.33 kg/m^2^ (range = 23-33 kg/m^2^) in the study population. Among female patients, the mean BMI was 28.75 ± 2.19 kg/m^2^ (range = 25-33 kg/m^2^), while among male patients, it was 27.43 ± 2.75 kg/m^2^ (range = 23-32 kg/m^2^) (p = 0.026) (Table 1).

**Table 1:** Demographic data. SD: standard deviation; BMI: body mass index

	Female	Male	P-value	Total
Age, mean ± SD (minimum-maximum)	66.95 ± 5.23 (57–76)	68.26 ± 4.79 (58–77)	0.286	67.96 ± 4.91 (57–77)
Dominant/Non-dominant (n)	46/22	14/7	0.428	60/29
BMI, mean ± SD (minimum-maximum)	28.75 ± 2.19 (25–33)	27.43 ± 2.75 (23–32)	0.026	28.44 ± 2.33 (23–33)
n	68	21		89

In the evaluation of CMS between the age groups (first group: seventh decade (n = 53); second group: eighth decade (n=32)), the preoperative mean CMS score in the first group was 27.55 ± 2.27 (range = 22-33), while in the second group, it was 24.56 ± 2.67 (range = 20-29). It was observed that the CMS score increased at each follow-up, and at the 12-month follow-up, the first group reached a mean of 69.43 ± 4.82 (range = 61-79), while the second group reached a mean of 64.09 ± 4.15 (range = 57-75) (all p < 0.001) (Table 2). Statistically, there was no significant difference between the third and fourth evaluations. However, significant differences were observed between the other evaluations (Table 3). Patients under the age of 60 (n = 4) were not included in the intergroup evaluations due to insufficient data. At the 12-month follow-up, male patients had a mean CMS score of 73.02 ± 4.11 (range = 65-79), while female patients had a mean CMS score of 65.93 ± 4.41 (range = 57-78), indicating a statistically significant difference (p < 0.001). In the comparative evaluation of the mean CMS scores at the 12-month follow-up between the original study [18] that provided the reference values and this study, it was observed that despite a significant improvement compared to the preoperative values in this study (152% increase in the first group and 161% increase in the second group; mean = 156%), the reference values could not be achieved (all p < 0.001) (Table 4). A weak negative correlation was observed between age and CMS at preoperative and 12-month follow-up assessments (r = -0.425 and -0.426, respectively). In addition, a very weak negative correlation was observed between BMI and CMS preoperatively, and a weak negative correlation was observed at the 12-month follow-up (r = -0.274 and -0.476, respectively) (Table 5).

**Table 2:** Comparison of CMS scores between the study groups. CMS: Constant-Murley scale; SD: standard deviation; first group: 61-70 years; second group: >70 years

	First group	Second group	P-value	Total
Preoperative, mean ± SD (minimum-maximum)	27.55 ± 2.27 (22–33)	24.56 ± 2.67 (20–29)	<0.001	26.45 ± 2.78 (20–33)
6th week, mean ± SD (minimum-maximum)	35.04 ± 2.53 (29–41)	32.84 ± 2.91 (27–39)	<0.001	34.27 ± 2.87 (27–41)
6th month, mean ± SD (minimum-maximum)	68.6 ± 4.71 (57–73)	63.22 ± 4.0 (57–73)	<0.001	66.72 ± 5.23 (57–78)
12th month, mean ± SD (minimum-maximum)	69.43 ± 4.82 (61–79)	64.09 ± 4.15 (57–75)	<0.001	67.59 ± 5.26 (57–79)
n	53	32		89

**Table 3:** Comparison of CMS scores between evaluations. CMS: Constant Murley scale; 1. eval: preoperative evaluation; 2. eval: sixth-week evaluation; 3. eval: sixth-month evaluation; 4. eval: 12th-month evaluation; SD: standard deviation; first group: 61-70 years; second Group: >70 years

	P-value		
	First group	Second group	Total
1.–2. eval	<0.001	<0.001	<0.001
2.–3. eval	<0.001	<0.001	<0.001
3.–4. eval	0.351	0.442	0.307

**Table 4:** Comparison of reference study values and current study 12th-month CMS scores. n1: current study number of patients; n2: reference study number of patients; CMS: Constant-Murley scale; SD: standard deviation; *summary independent-sample t-test; first group: 61-70 years; second group: >70 years

		Current study, mean ± SD	Reference study, mean ± SD	P-value*
Male	First group (n1 = 13, n2 = 127)	73.85 ± 4.26	90 ± 6	<0.001
	Second group (n1 = 6, n2 = 33)	70.33 ± 3.33	86 ± 7	<0.001
Female	First Group (n1 = 40, n2 = 91)	68 ± 4.1	82 ± 5	<0.001
	Second group (n1 = 26, n2 = 50)	62.65 ± 2.76	81 ± 4	<0.001

**Table 5:** Correlation of CMS and SF-36 values with age and body mass index. CMS: Constant-Murley scale; SF-36: Short Form-36; BMI: body mass index

			Age		BMI	
			r-value	Interpretation (correlation)	r-value	Interpretation (correlation)
CMS		Preoperative	-0.425	Weak	-0.274	Very weak
		12th month	-0.426	Weak	-0.476	Weak
SF-36	Physical components, average	Preoperative	-0.621	Moderate	-0.189	Very weak
		12th month	-0.688	Moderate	-0.174	Very weak
	Mental components, average	Preoperative	-0.424	Weak	-0.134	Very weak
		12th month	-0.351	Weak	-0.117	Very weak

When examining the SF-36 values, significant improvements were observed in physical functioning, physical role, bodily pain, general health, mental health, and emotional role in the 12-month follow-up compared to preoperative values (all p < 0.001). However, there was no significant difference in social functioning and vitality values (p = 0.099 and 0.255, respectively) (Table 6). When compared to the reference values for the Turkish population [20], it was determined that, in the first group (age <65 years), all preoperative values, except for vitality, and in the second group (age ≥65 years), in addition to vitality, physical functioning, and social functioning scores, were at a significantly lower level. When comparing the postoperative values, it was observed that the statistical differences in all parameters, except for physical functioning, physical role, and social functioning in the first group, were no longer present. However, it was observed that these values ​​also increased compared to the preoperative period (Table 7). It was observed that there were weak and moderate negative correlations between SF-36 and age (r = -0.621, -0.688, -0.424, -0.351), and a very weak negative correlation with body mass index (r = -0.189, -0.174, -0.134, -0.117), but there were no significant differences between preoperative and postoperative values (Table 5).

**Table 6:** Change of SF-36 values between preoperative and 12th month (all patients). SD: standard deviation; SF-36: Short Form-36; PF: physical functioning; RP: physical role; BP: bodily pain; GH: general health; MH: mental health; RE: emotional role; SF: social functioning; VT: vitality

		Preoperative	12th month	P-value
Physical components (mean±SD)	PF	55.41 ± 9.92	60.83 ± 8.58	<0.001
	RP	54.92 ± 9.64	66.88 ± 6.79	<0.001
	BP	63.1 ± 5.7	70.35 ± 10.1	<0.001
	GH	56.4 ± 9.74	64.2 ± 4.28	<0.001
Mental components (mean±SD)	MH	65.28 ± 4.25	71.74 ± 2.12	<0.001
	RE	76.3 ± 4.53	86.46 ± 5.02	<0.001
	SF	80.8 ± 4.16	81.7 ± 4.71	0.099
	VT	61.85 ± 4.47	62.68 ± 4.51	0.255

**Table 7:** Comparison of the SF-36 values of the current study groups and the reference study. SF-36: Short Form-36; SD: standard deviation; first group: <65 years; second group: ≥65 years; *: summary independent-sample t-test; PF: physical functioning; RP: physical role; BP: bodily pain; GH: general health; MH: mental health; RE: emotional role; SF: social functioning; VT: vitality

		First group	Reference	P-value*	Second group	Reference	P-value*
PF (mean ± SD)	Preoperative	69.29 ± 3.52	81.3 ±2 5.5	0.017	49.05 ± 2.77	56.3 ± 37.2	0.131
	12th month	71.5 ± 3.85		0.043	55.93 ± 4.88		0.939
RP (mean ± SD)	Preoperative	68.36 ± 3.67	86.8 ± 32.3	0.004	48.75 ± 2.69	64 ± 46.4	0.011
	12th month	73.89 ± 3.94		0.035	63.66 ± 5.21		0.955
BP (mean ± SD)	Preoperative	69.68 ± 3.28	83.6 ± 21.4	0.001	60.08 ± 3.65	70.3 ± 25.9	0.003
	12th month	84.04 ± 3.17		0.809	64.07 ± 4.1		0.064
GH (mean ± SD)	Preoperative	59.04 ± 3.66	70.8 ± 18.3	0.001	55.2 ± 3.13	62.8 ± 20.7	0.005
	12th month	68.86 ± 2.38		0.576	62.07 ± 3.09		0.785
MH (mean ± SD)	Preoperative	66.75 ± 2.73	73.5 ± 11.5	0.003	64.61 ± 4.66	72 ± 14.8	<0.001>
	12th month	72.36 ± 2.34		0.601	71.46 ± 1.96		0.777
RE (mean ± SD)	Preoperative	80.89 ± 3.5	95 ± 20.5	0.001	74.21 ± 3.22	86.5 ± 32.5	0.004
	12th month	92.4 ± 2.57		0.288	83.74 ± 3.17		0.510
SF (mean ± SD)	Preoperative	82.57 ± 4.42	94.7 ± 12.9	<0.001	79.87 ± 2.53	83.7 ± 24.4	0.224
	12th month	84.75 ± 6.11		<0.001	80.3 ± 3.05		0.281
VT (mean ± SD)	Preoperative	65.29 ± 3.22	65.7 ± 14.1	0.882	60.28 ± 4.08	60.9 ± 15.8	0.764
	12th month	66.21 ± 3.35		0.849	61.07 ± 4.04		0.934
n		28	446		61	116	

In the post-hoc power analyses performed after our retrospective comparative study, in which a total of 89 patients were evaluated, the effect value was 1.18, α-error was 0.05, and power (1-β-error) was 0.99.

## Discussion

RSA is frequently employed as a therapeutic approach for the management of conditions such as massive rotator cuff tears, RCTA, and traumatic shoulder injuries [2]. In this study, which investigated the functional outcomes and quality of life of patients who underwent RSA due to RCTA, it was determined that the functional assessment criterion, CMS, did not reach the normal shoulder reference values at the 12th month after the surgery. However, a significant increase of 156% (p < 0.001) was observed compared to the preoperative values. The lack of Turkish population-specific reference values for the CMS used in this study may have negatively impacted the interpretation of the results. In the assessment of the quality of life using the SF-36 evaluation, statistically significant improvement was observed in all parameters other than social functioning (p = 0.099) and vitality (p = 0.255) (all p<0.001). It was determined that all parameters except physical functioning, physical role, and social functioning in patients under 65 years of age reached the reference values ​​statistically.

Castricini et al. [16] identified a statistically significant improvement in CMS (from 23 to 66) (p < 0.001) between the preoperative and postoperative stages in their study, which consisted of a series of 80 patients, including 13 who underwent RSA due to RCTA, similar to our study. It was observed that there was no preoperative assessment for SF-36, but when compared to the normal population after the operation, there was no statistically significant difference. In this study, which investigated 89 patients with RCTA, a similar pattern was observed. The CMS score increased from 26.45 to 67.59 (p < 0.001). In the SF-36 assessment, it was observed that for patients under the age of 65 years, there was no statistically significant difference between the reference values and the parameters other than physical functioning, physical role, and social functioning. A comparison was done between the patient group aged 45-64 years in the study that provided the reference values [20] and patients under the age of 65 years in this study. However, it is believed that the absence of patients under the age of 57 in this study could have a negative impact on the results.

Sabesan et al. [15] evaluated the three most used shoulder scales in a series of 140 patients undergoing RSA. They reported that the CMS had the strongest correlation with arm function compared to the others (American Shoulder and Elbow Surgeons scores and subjective shoulder values). In the same study, it was shown that the mean value of the CMS increased from 26.64 preoperatively to 59 postoperatively. In this study, which utilized CMS for functional evaluation, the preoperative scores showed similarity to the mentioned study (26.45 ± 2.78). However, it was observed that the score in the 12th month postoperatively increased relatively more, reaching 67.59 ± 5.26. In another study, Imam et al. [21] demonstrated that in a series of 159 patients (including 108 with RCTA), the preoperative CMS value of 28.2 ± 13.3 increased to 75.5 ± 22.8 (p < 0.0001) postoperatively. Additionally, Favard et al. [22] showed that the preoperative CMS score of 24 increased to 66 after the operation.

In the study conducted by Rauck et al. [11], which included measurements of SF-36 in 10 patients, a statistically significant improvement was observed in physical functioning and physical role between preoperative and two-year postoperative comparisons. However, no significant changes were found in the other parameters of SF-36 (p=0.014, 0.017, respectively). In the current study, in addition to improvements in physical functioning and physical role, significant improvements were also observed in scores of bodily pain, general health, mental health, and emotional role (all p<0.001). There was an increase in social functioning and vitality values, but no statistically significant difference was found (p = 0.099 and 0.255, respectively).

In the study conducted by Nascimento et al [8]. on a series of 20 patients who underwent RSA due to RCTA, the postoperative SF-36 value was determined to be 63 by calculating the arithmetic mean of eight parameters. In this study, when the arithmetic mean was calculated, the SF-36 value was 70.6. We consider that using the SF-36 value, which should be calculated by separately evaluating all parameters as standard, in this manner is not appropriate.

Reid et al. [23] demonstrated that an increase in BMI in anatomical shoulder arthroplasty and RSA has a negative impact on postoperative functional scores. In this study, a very weak negative correlation was observed between CMS and BMI preoperatively (r = -0.274). At the 12-month follow-up, a weak negative correlation was observed (r = -0.476). This finding indicates a correlation between BMI and postoperative functional outcomes in line with the study that highlighted the impact of BMI on postoperative functional outcomes.

This study has several limitations. The most important aspect is the retrospective nature of the study. Another limitation is the absence of third-month values in the CMS assessment and the absence of sixth-week, third-month, and sixth-month values in the SF-36 assessment. Although the inclusion of 89 patients who underwent RSA for RCTA can be considered substantial, we are of the opinion that these limitations can be overcome through high-powered prospective studies. In addition, we believe that the normal values of functional parameters and quality of life ​​may be different for each community. Although a comparison was made for SF-36 according to social reference values ​​in this study, the fact that the reference values ​​used for CMS do not belong to Turkish society may have led to a bias. We believe that there is a need for a study reporting social reference values ​​for CMS.

## Conclusions

It has been observed that RSA performed due to RCTA improves functional outcomes and quality of life. RSA is a good option in case of appropriate indication. Although there was a mean 156% increase in CMS after surgery compared to preoperatively, it was observed that the normal shoulder CMS reference values reported in the literature ​​could not be reached. We think that this may be due to the lack of social reference values ​​in the study. However, it was observed that the social shoulder reference values ​​for SF-36 could be reached after an RSA operation, especially in patients over the age of 65 years.
